# Demyelination as a harbinger of lymphoma: a case report and review of primary central nervous system lymphoma preceded by multifocal sentinel demyelination

**DOI:** 10.1186/s12883-016-0596-1

**Published:** 2016-05-21

**Authors:** Mark D. Kvarta, Deva Sharma, Rudolph J. Castellani, Robert E. Morales, Stephen G. Reich, Amy S. Kimball, Robert K. Shin

**Affiliations:** Program in Neuroscience and Medical Scientist Training Program, University of Maryland School of Medicine, Baltimore, Maryland; Departments of Physiology, University of Maryland School of Medicine, Baltimore, Maryland USA; Department of Pathology, University of Maryland School of Medicine, Baltimore, Maryland USA; Department of Diagnostic Radiology, University of Maryland School of Medicine, Baltimore, Maryland USA; Department of Neurology, University of Maryland School of Medicine, Baltimore, Maryland USA; Department of Internal Medicine, University of Maryland School of Medicine, Baltimore, Maryland USA; Vascular Medicine Institute, University of Pittsburgh Medical Center, Pittsburgh, Pennsylvania USA; Department of Neurology, MedStar Georgetown University Hospital, District of Columbia, Washington USA

**Keywords:** Primary CNS Lymphoma, Demyelination, Multiple Sclerosis, Pre-operative steroids

## Abstract

**Background:**

Primary central nervous system lymphoma (PCNSL) may rarely be preceded by “sentinel demyelination,” a pathologic entity characterized by histologically confirmed demyelinating inflammatory brain lesions that mimic multiple sclerosis (MS) or acute disseminated encephalomyelitis (ADEM). Interpreting the overlapping radiologic and clinical characteristics associated with each of these conditions—contrast-enhancing demyelination of white matter and relapsing and remitting steroid-responsive symptoms respectively—can be a significant diagnostic challenge.

**Case presentation:**

We describe a 57-year-old woman with an unusual clinical course who presented with multi-focal enhancing white matter lesions demonstrated to be inflammatory demyelination by brain biopsy. Despite a good initial response to steroids and rituximab for treatment of presumed tumefactive multiple sclerosis, the patient’s condition rapidly deteriorated, and a repeat brain biopsy six months later was consistent with a diagnosis of diffuse large B-cell lymphoma.

**Conclusions:**

Early clinical suspicion for PCNSL and awareness that biopsied lesions may initially show sentinel demyelination suggestive of alternate diagnoses may be essential for early initiation of appropriate therapies and mitigation of disease progression. Clinical, pathophysiological, and diagnostic aspects of sentinel demyelination and PCNSL are discussed.

## Background

Primary central nervous system lymphomas (PCNSL) are rare neoplasms that account for less than 1 % of all brain tumors [1, 2]. Though more common in the setting of immunosuppression, the incidence of PCNSL has increased in the last three decades among immunocompetent individuals [1–4]. PCNSLs are most frequently diffuse large B-cell non-Hodgkin lymphomas, represent 4 % of intracranial neoplasms, and present at a median age of 60 [5]. Surface expression of both BCL6 and IRF4, along with immunoglobulin gene rearrangement in the majority of PCNSL B-cells indicate that the tumor cells have an activated B-cell phenotype [6].

PCNSL rarely presents with steroid-responsive, multifocal demyelinating “sentinel” lesions characterized by a predominance of T-cell infiltrates and few B-cells [1–4]. These demyelinating brain lesions may be histologically indistinguishable from those seen in multiple sclerosis (MS). Because both MS and PCNSL may present with contrast-enhancing white matter lesions and relapsing and remitting symptoms and signs that improve with steroid therapy, this can lead to diagnostic confusion. Biopsy of these sentinel lesions early in the course of disease, even months before PCNSL is ultimately diagnosed, can lead to diagnostic confusion, especially in the setting of preceding corticosteroid administration [1, 2, 7, 8].

Here we describe a case of a steroid-responsive, histologically-confirmed inflammatory demyelinating lesion which proved later to be a rapidly progressing PCNSL diagnosed on repeat biopsy. We offer guidelines to the approach to a brain biopsy in older adults, to facilitate increased diagnostic accuracy.

## Case presentation

In October 2009, a previously healthy 57-year-old woman presented with a six week history of progressive fatigue, confusion and headache associated with a right inferior homonymous quadrantanopsia. The patient reported frequently bumping into objects as well as several recent falls. She denied alcohol or drug use and was not taking any prescribed medications. Physical exam revealed a mild right hemiparesis and a wide-based gait, in addition to the visual field defect. There was no relevant past medical, family, or psychosocial history. The patient’s clinical timeline is summarized in Table 1.

**Table 1:** Clinical course timeline

Date	Event
October 10–15, 2009	A 57 year-old woman with no significant past medical history presents with 6 weeks of fatigue, confusion and headache, with physical exam revealing right inferior homonymous quadrantanopsia, mild right hemiparesis and a wide-based gait admitted for evaluation of multiple brain lesions
	Brain MRI revealed multiple enhancing lesions, including right frontal and left parieto-occipital mass lesions concerning for neoplasm
	Patient was treated with pre-operative corticosteroids for 3 days
October 28	Biopsy of the left parieto-occipital lesion revealed mononuclear infiltrates with evidence of demyelination, but without evidence of malignancy
	Patient was treated with a second round of intravenous corticosteroids for presumed acute disseminated encephalomyelitis or tumefactive multiple sclerosis
	Patient was discharged home after showing rapid clinical improvement
November 23	Over the course of several weeks, the patient developed progressive dysarthria and right-sided weakness, prompting readmission to the hospital
	A repeat brain MRI showed an increase in the size of the left-sided lesions, with vasogenic edema and subfalcine herniation
November 25	Patient refused a repeat brain biopsy
November 27	Patient was treated with a third round of intravenous corticosteroids and rituximab infusions, resulting in symptomatic improvement and discharge from the hospital
April 13, 2010	Over the following five months, after initial improvement, the patient again developed progressively worsening right hemiparesis and dysarthria, resulting in a second hospital readmission
	MRI of the brain showed enlargement of the prior intracranial lesions with nodular enhancement suggestive of neoplasm
May 25	A second brain biopsy of the left parietal lesion was consistent with diffuse large B-cell lymphoma
	Patient was treated with high dose methotrexate and leucovorin
June 7	In light of her continued clinical deterioration despite treatment, the patient was transferred to hospice care
	Patient passed away in hospice care

**Fig. 1:**
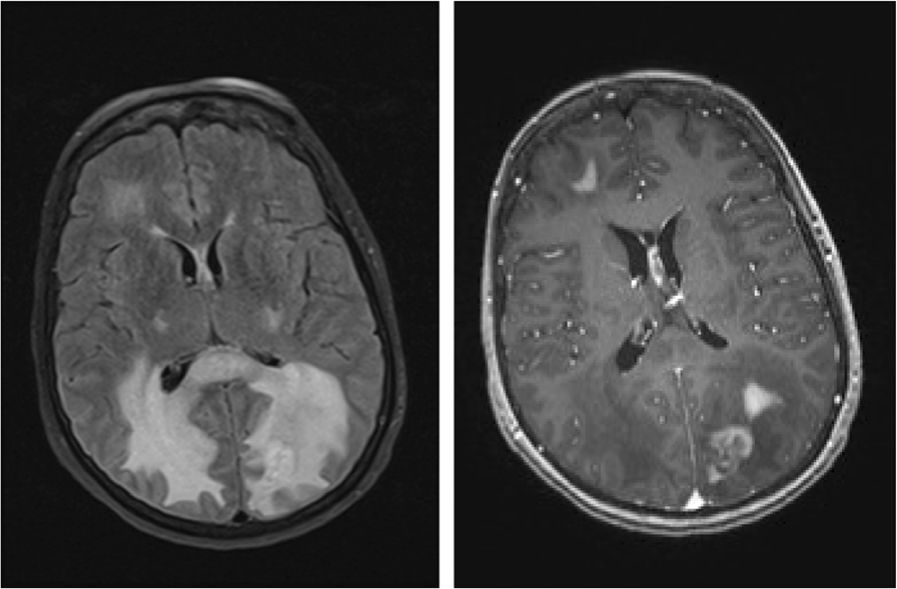
Brain MRI (October 2009): axial FLAIR (left) and axial T1 post contrast (right) revealing right frontal and left parieto-occipital enhancing lesions with surrounding edema

A brain MRI revealed multiple enhancing lesions, including right frontal and left parieto-occipital mass lesions, that are nonspecific but concerning for demyelination, an infectious or inflammatory process, or neoplasm (Fig. 1).

**Fig. 2:**
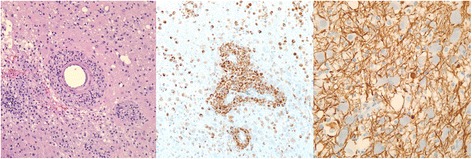
Histopathology (October 2009): H&E 60x showing reactive perivascular astrocytosis and macrophages (left), CD68 60x immunostain for histiocytes (center), NF 180x showing relative preservation of axons (right)

Following pre-operative corticosteroid treatment (dexamethasone 6 mg IV q6h for 3 days), the left parieto-occipital lesion was biopsied (Fig. 2), revealing mononuclear infiltrates with numerous T cells (CD3+), histiocytes (CD68+), and a few scattered B cells (CD20+). There was no cytological evidence of malignancy, and axonal preservation was evident, consistent with demyelination. TAF, GMS, AFB and FITE stains did not demonstrate any microorganisms. In addition, HSV 1 and 2 immunohistochemistry, EBV in-situ hybridization and HIV serologies were negative.

The patient was diagnosed with acute multi-focal demyelination, presumed to be acute disseminated encephalomyelitis or tumefactive multiple sclerosis, and treated with intravenous corticosteroids. She improved rapidly but shortly after discharge developed worsening right-sided weakness and dysarthria and returned several weeks later. At that time, she was alert but mildly disoriented, with intact sensation to temperature, pinprick and vibration, brisk deep tendon reflexes throughout, and bilateral ankle clonus. Gait again was wide-based and unsteady.

**Fig. 3:**
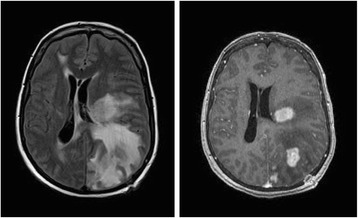
Brain MRI (November 2009): axial FLAIR (left), axial T1 post contrast (right) demonstrating an increase in the size of the left-sided lesions, vasogenic edema, and mass effect leading to subfalcine herniation (notice left parietal burr hole from the biopsy)

**Fig. 4:**
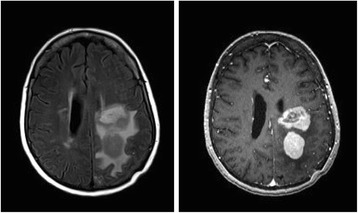
Brain MRI (April 2010): axial FLAIR (left), axial T1 post contrast (right) reveal progression of nodular enhancement of the persisting left-sided lesions

**Fig. 5:**
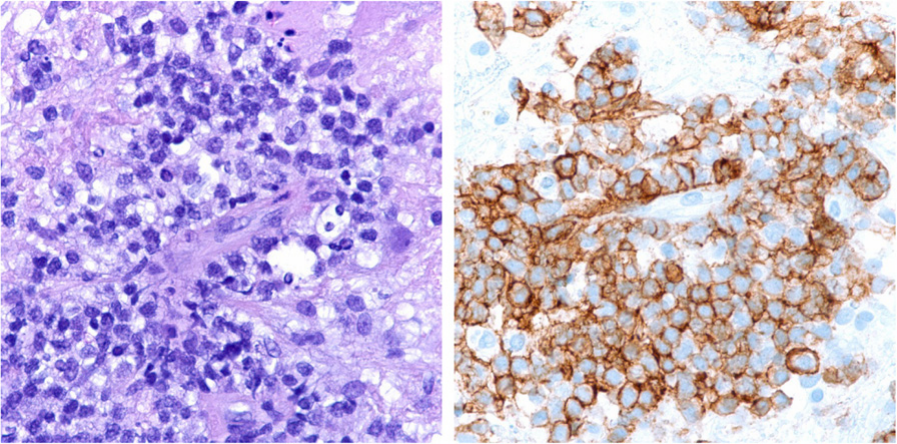
Histopathology (May 2010): H&E 180x showing large malignant perivascular lymphoid cells (left), CD20 180x pan B-cell immunostain (right)

Repeat MRI showed decreased edema and enhancement of the right-sided lesions, but an increase in the size of the left-sided lesions with vasogenic edema and subfalcine herniation (Fig. 3). The patient refused a repeat brain biopsy. The symptoms, signs and radiographic lesions improved with a second course of intravenous corticosteroids and four weekly rituximab infusions. She remained clinically stable for the next five months, but was ultimately hospitalized for a third time with worsening right hemiparesis and dysarthria. MRI showed enlargement of the prior intracranial lesions with nodular enhancement strongly suggestive of a neoplasm (Fig. 4). PET-CT scan revealed increased metabolism within the brain lesions without evidence of extracranial malignancy. A second brain biopsy of the left parietal lesion was obtained through the previous burr hole. Six months after the first biopsy of the same lesion, the second biopsy revealed markedly different histopathologic results (Fig. 5), including large, perivascular malignant lymphoid cells uniformly positive for CD20 with a smaller number of CD3+ mature T cells – consistent with diffuse large B-cell lymphoma. Despite treatment with high-dose methotrexate and leucovorin, the patient’s condition deteriorated. She was transferred to a hospice care facility and died several days later.

## Discussion

As this case demonstrates, PCNSL may be preceded by demyelinating lesions [1–4, 7] suggestive of tumefactive multiple sclerosis or ADEM. PCNSL may mimic MS with waxing and waning brain lesions; PCNSL symptoms and signs are generally responsive to corticosteroids [1]. Although MS is typically characterized by multiple small demarcated plaques, tumefactive MS and other atypical forms of MS can present radiologically as space-occupying lesions with mass effect and edema resembling brain tumors [9]. In this patient, two brain biopsies from the same location yielded significantly different results. The first revealed numerous CD3+ infiltrates, scattered CD20+ immunoreactive cells, and axonal sparing and demyelination without evidence of a neoplasm. The second biopsy revealed large malignant perivascular lymphoid cells and a striking increase in CD20+ immunoreactivity diagnostic of PCNSL. *How did two biopsies of the same lesion, separated only by months, yield such different histopathological results?*

### Randomly coincidental findings

Could the co-existence of “sentinel demyelination” and PCNSL be simply coincidental and unrelated pathologies? Pure coincidence has been hypothesized when MS and PCNSL have been found in two different locations in the same patient separated in time by many years [10]. In our case, however, both pathologies were found within the same lesion within a six month time interval, making coincidence seem less likely.

### Missed first biopsy

Is it possible that the first biopsy missed the "true" lesion harboring malignant B-cell clones? If so, how do we explain the presence of inflammatory demyelination? A paraneoplastic phenomenon is possible, as anti-MOG antibodies were found in the sera of a patient with sentinel demyelinating lesions preceding PCNSL [2]. These may have been produced by a monocolonal population of transformed B-cells, promoting autoimmune demyelination. Although considerable T-cell infiltration is seen in PCNSL [11], a halo of T-cell infiltration and demyelination *surrounding* lymphoma has never been described to our knowledge.

### Malignant transformation during latent period

Could tumefactive demyelination have transformed into B-cell lymphoma [2]? In our patient, the radiographic appearance of the lesion changed in conjunction with the biopsy results, suggesting that the lesion itself may have histologically evolved. It has been hypothesized that lymphocytes may become entrapped in the brain following an inflammatory response and may later undergo malignant transformation [2, 12]. However, patients with inflammatory diseases of the CNS have not been shown to have an increased incidence of PCNSL [13], although there is a well-described increased incidence of lymphoma in patients with systemic inflammatory diseases [14].

### Disruption of anti-tumor immune response by corticosteroids

Could the T-cell infiltrates in the initial biopsy have represented a cell-mediated immune response against the lymphoma, thereby masking a diagnosis of PCNSL [4, 11]? This hypothesis would predict that when the host immune system is disrupted by intermittent or prolonged corticosteroid therapy, a “suppressed” neoplastic B-cell clone could emerge from lymphocytic infiltrates [15].

The observation that the presence of non-malignant infiltrates consisting predominantly of T-cells correlates with improved survival in follicular lymphoma and reports of spontaneous regression of lymphoma in immunocompetent individuals support the existence of a suppressive cell-mediated anti-tumor response [16–18].

### Masking of diagnosis by corticosteroids

Did treatment with corticosteroids mask the presence of malignant B cells? Many B-cell lymphomas, including PCNSL, are steroid-responsive [2–4, 17, 19, 20], whereas activated T-cells may be relatively protected from glucocorticoid-induced apoptosis [11, 17]. Selective survival of a few steroid-resistant B-cell clones following corticosteroid administration could explain the “missing” B-cells on the initial biopsy, diminishing steroid responsiveness over time, and ultimately, the emergence of steroid-resistant B-cell infiltrates on repeat biopsy. A recent retrospective study examining approximately 1000 cases of PCNSL suggested that the effects of corticosteroid treatment preceding biopsy rendered accurate diagnosis from biopsy impossible in *up to 50 % of cases* [8].

### Differentiating between demyelination and CNS lymphoma

Summarizing 15 cases from the literature, combined with our own case presentation, Table 2 details patient demographics and presenting signs and symptoms of patients who were initially suspected of a demyelinating disease but were later diagnosed with primary CNS lymphoma.

**Table 2:** 16 patients with evidence of demyelination, ultimately diagnosed with CNS lymphoma

Mean Age	46.2 (range 20–65)
Female:Male	12:4
Initial response to steroids	16/16 (100 %)
Mean time from initial presentation to lymphoma diagnosis	23.2 months (range 6–65)
Symptoms & signs	
Hemiparesis	50 % *
Visual symptoms (diplopia, anopsia or visual field cut, nystagmus)	50 % *
Cognitive (memory, concentration, confusion)	50 % *
Ataxia or gait disturbance	40 % *
Dysarthria	31.3 % *
Headache	31.3 % *
Fatigue or somnolence	25 % *
Vertigo	25 %
Numbness or paresthesia	25 %
Seizures	25 %
Vomiting	12.5%
Pain	12.5%
Incontinence	6.3%
Anorexia	6.3%
Hearing loss	6.3%
*-our patient (Cases referenced: [1, 2, 4, 10, 14, 22, 33–37])	

While each patient presentation is unique, there are some clinical “red flags” that should increase suspicion for PCNSL (Table 3). Examples include worsening in radiographic appearance of brain lesions over time, deteriorating clinical course despite adequate treatment for demyelinating disease, sustained clinical dependence on corticosteroids (which is unusual in MS) [21], and advanced age. On average, it has previously been reported that immunocompetent patients with PCNSL present between the ages of 55–70, while patients with MS and ADEM most frequently present in young adulthood and childhood respectively [22]. In addition, older patients with MS are more likely to have spinal cord involvement (80 % in MS vs. <2 % in PCNSL) and CSF oligoclonal bands (98 % in MS vs. only 27 % in PCNSL) when compared to patients with PCNSL [7].

**Table 3:** Criteria which should raise clinical suspicion for PCNSL and *sentinel demyelination* in patients with white matter lesions

Clinical	• Middle to older age with no prior clinical episodes or radiographic lesions suggestive of MS • Rapidly deteriorating course • Steroid dependence • Lack of spinal cord involvement
Imaging	• Increased enhancement or lesion size over time • Disproportionate mass effect
CSF	• Abnormal cytology (clonal IgG gene rearrangement) • No oligoclonal bands

Barkhof and modified McDonald criteria can be used to predict the risk of progression from a clinically isolated syndrome (CIS) or ADEM to MS [23, 24]. Identifying the predictive value of spatial distribution criteria for the development of PCNSL from a single sentinel demyelinating event would be extremely relevant and may shed further light on the pathophysiology of this disease progression. In our patient, lesions involved posterior frontal deep white matter, the left parietal lobe, and bilateral occipital lobe with callosal involvement. MS lesions are common in these areas. ADEM lesions also include periventricular and subcortical white matter, and often involve corpus callosum, thalamus, and basal ganglia [24]. Discerning radiologically between these entities will remain challenging without identification of more specific patterns of imaging findings.

Thorough approaches to both radiographic and histopathologic differential diagnoses in such cases have been detailed elsewhere [19, 20, 25]. Radiographically tumefactive MS is frequently associated with ring- or heterogenous enhancement, with features of varying levels of enhancement and pallor reflecting evolution of lesions over time. However, ring, homogenous and heterogenous gadolinium patterns have all been observed in large case series [26]. Ring-enhancement and T1-hypointensities (‘black holes’) are associated with persistence and severity [27–30]. PCNSL, in contrast, is most commonly diffusely enhancing due to lack of central necrosis [19], as in this patient’s early images.

If a brain biopsy is being considered in this context of initial diagnostic workup, steroids should be withheld, unless rapid neurological deterioration is present, as most patients with PCNSL tolerate deferral of steroid therapy as long as biopsy is performed in a timely manner [20]. However, if steroid treatment has already been initiated and MRI is consistent with lymphoma, empirical treatment for PCNSL in the absence of histopathologic confirmation of disease may be cautiously considered, as withdrawal of steroids with plans for re-biopsy has often resulted in poor patient prognosis and rapid death [31].

## Conclusions

This case serves as a reminder that PCNSL can be preceded by sentinel lesions indistinguishable from the demyelination of MS or ADEM, and that steroid treatment before biopsy obscures a diagnosis of PCNSL. Clinicians must be vigilant to realize that not all histologic and radiologic evidence of demyelination correspond to a primary demyelinating disease. It is important to maintain a high level of suspicion for PCNSL to facilitate early diagnosis and treatment, particularly with specific clinical and diagnostic features (Table 3). The combination of chemotherapy and radiotherapy has significantly improved prognosis for patients with PCNSL, leading to prolonged survival and even cure in some cases [21, 32]. Hence, early diagnosis of PCNSL will ultimately lead to more timely therapeutic interventions. Finally, we would recommend discretion in the use of corticosteroids prior to biopsy in patients in whom PCNSL is suspected.

## Ethics and consent to participate

Not applicable for a case report on this patient that was treated without a research intention. This case report does not meet the definition of human research by the United States Department of Health and Human Services or the Food and Drug Administration guidelines. This is also in accordance with the ethical manual and guidelines of the World Medical Association (see 59^th^ WMA General Assembly, Seoul, Republic of Korea, October 2008).

## Consent to publish

Written informed consent was obtained from the next of kin of the patient for publication of this case report and accompanying images. A copy of the written consent is available for review by the editor of this journal.

## Availability of data and materials

All data supporting our findings are contained within the manuscript.

## Abbreviations

ADEM: acute disseminated encephalomyelitis
CNS: central nervous system
MS: multiple sclerosis
PCNSL: primary central nervous system lymphoma
